# High grade uterine adenosarcoma with sarcomatous overgrowth in a young woman amenable to primary surgical reduction: A case study and literature review

**DOI:** 10.1016/j.gore.2021.100920

**Published:** 2021-12-29

**Authors:** D.D. Dowding, L.M. Wayne, A.S. Guirguis

**Affiliations:** a A.T. Still University – Kirksville College of Medicine, Kirksville, MO 63501, United States; b Gynecologic Cancer Institute of Chicago, Oak Lawn, IL 60453, United States

**Keywords:** Uterus, Adenosarcoma, Sarcomatous overgrowth, Rhabdomyosarcomatous differentiation, Heterologous, Surgical reduction

## Abstract

Mullerian adenosarcomas are rare and often low-grade mixed tumors that typically respond well to optimal surgical resection. However, adenosarcoma with sarcomatous overgrowth (ASSO) is a high-grade mixed tumor commonly associated with invasion, metastasis, and a poor prognosis. The health care providers herein report a case study of a patient diagnosed with ASSO who has maintained remission status for 19 months following radical surgical resection alone. The patient, a 24-year-old Caucasian female without significant medical history, initially complained of abdominal fullness, pelvic pressure, altered menses, and unintentional weight loss. A necrotic cervical mass was present on the exam; mass biopsy revealed spindle cell sarcoma with rhabdomyosarcomatous differentiation. The patient underwent exploratory laparotomy, total abdominal hysterectomy, bilateral salpingectomy, radical tumor debulking, and pelvic and periaortic lymph node dissection. Histopathological diagnosis of the resected specimen was consistent with ASSO, limited to 0.7 cm out of 2.0 cm of myometrial thickness, with negative lymph node and parametrial tissue, consistent with Stage IB disease. She did not receive adjuvant chemotherapy or radiation and has remained disease-free to date. Because of the rarity of ASSO and lack of abundant case study reports, uniform clinical guidelines for treatment following surgical resection of a high-grade adenosarcoma remain unclear. However, the case study below may suggest that radical surgical debulking of this disease with negative margins in young patients with early-stage disease can be sufficient in treating high-grade ASSO, despite their typical aggressive nature.

## Highlights

• Uterine ASSO is an exceedingly rare and aggressive disease in young females.
• There are no current guidelines for treatment of uterine ASSO.
• Tumor debulking with close observation alone is reasonable in early stage disease.

## 1 Case presentation

**Fig. 1:**
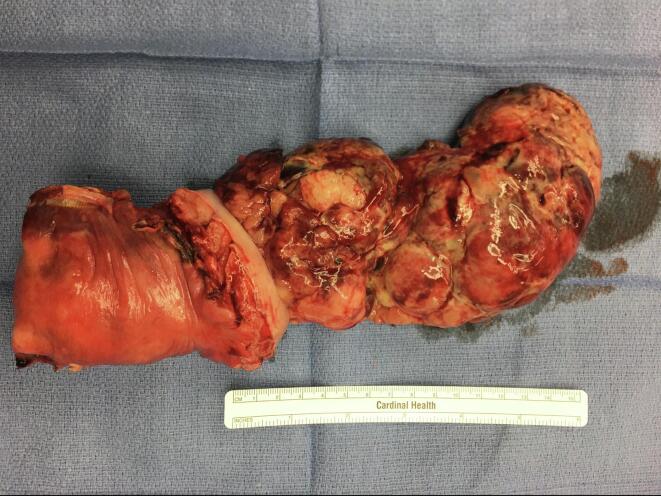
A uniform fleshy and partially necrotic mass extending through the cervical canal and into the vagina measuring about 16 cm from the cervical os.

**Fig. 2:**
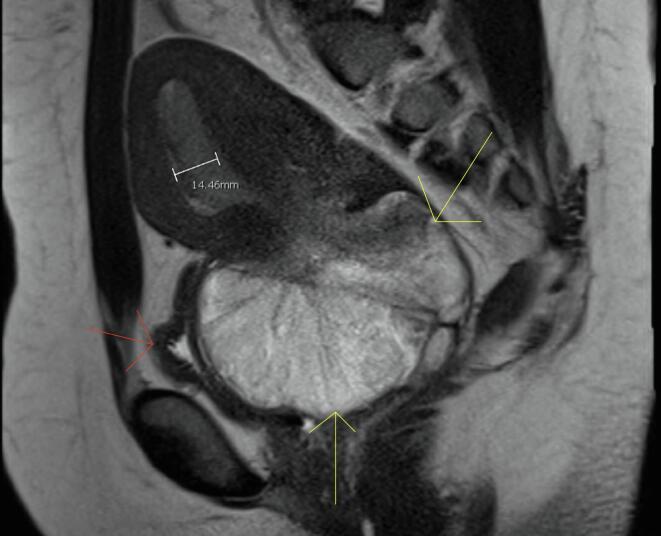
T2 weighted image of the large ill-defined mass in situ as outlined by the yellow arrows, with the bladder displaced anteriorly noted by the red arrow. (For interpretation of the references to colour in this figure legend, the reader is referred to the web version of this article.)

A 24-year-old nulligravid female with no significant past medical history presented with complaints of abdominal fullness and persistent pelvic pressure for one month. She also noted altered menses with new-onset menorrhagia, difficulty with urination, and unintentional weight loss of 25 lb over the preceding 8 months. The patient's OB-GYN history was notable for menarche at 12 years old; the typical cycle length was 28 days with 7 days of bleeding. Her medication list included Nortrel 1/35 without side effects. Pelvic examination revealed a black, necrotic mass with foul-smelling yellow discharge in the vaginal canal (Fig. 1). MRI of the pelvis noted 9.1 × 8.7 × 9.1 cm ovoid heterogeneous predominantly high T2 signal mass at the cervix/lower uterus with ill-defined extension into the myometrium at the posterior aspect of the mid uterus and distortion of the endometrium. There was also a prominent left external iliac lymph node (Fig. 2).

A mass biopsy resulted in spindle cell sarcoma with rhabdomyosarcomatous differentiation. Mayo Clinic concurred with this diagnosis, noting that it was unclear if this was a pure sarcoma or part of a biphasic malignancy. Cleveland Clinic then reviewed the pathology slides and reported atypical spindle cells, suspicious for sarcoma. Spindle cells were hyperchromatic, along with scattered bizarre tumor cells showing frequent mitotic activity. Spindle cells were negative for desmin, CD10, myogenin, ALK1, and WT1. There was diffuse strong positivity for p16 and patchy strong expression for MyoD1, supportive of rhabdomyosarcomatous differentiation. Ki67 labeling index was approximately 80%. Features and staining increased suspicion for underlying sarcoma of some sort; however, the sample was too small for definitive diagnosis.

The surgeons subsequently performed an exploratory laparotomy, total abdominal hysterectomy, bilateral salpingectomy, radical tumor debulking, staging, pelvic and periaortic lymph node dissection, bilateral ureterolysis, cystoscopy, and bilateral ureteral stent insertion. Intraoperatively, the ovaries appeared to be completely within normal limits and were left in situ. A complete lymphadenectomy was also performed given suspicious findings on PET and MRI, some lymph nodes were noted to be enlarged intraoperatively but this appeared reactive in nature. The lower uterine segment and cervix measured at least 11 cm in diameter and approximately 15 cm in length. The uterus and cervix were removed and a partial radical vaginectomy was performed without transecting the tumor. The final surgical pathology was consistent with adenosarcoma with sarcomatous overgrowth (ASSO) from the posterior endometrium, approximately 21 cm in greatest dimension, including intrauterine and extrauterine components. There was 0.7 cm invasion out of 2.0 cm myometrial thickness. Bilateral parametria, lymphovascular space invasion (LVSI), pelvic washings, and 9/9 lymph nodes were negative for malignancy. The ER and PR testing were both < 1% staining. Mayo Clinic reviewed the final pathology; their findings were consistent with stage IB high-grade Mullerian ASSO.

Given the patient's young age and lack of clear benefit of adjuvant chemotherapy, the patient was routinely followed with alternating CT chest/abdomen/pelvis and PET every 3 months post-operatively. The patient had a questionable recurrence in the lung six months post-operatively on routine PET. Fine needle aspiration was performed, and there was a concern for metastatic disease of Mullerian origin. However, malignancy was ruled out on final lung wedge resection pathology. At present, 19 months after her initial surgery, the patient remains in remission and continues to undergo routine surveillance with imaging and physical exams.

## 2 Literature review

Uterine adenosarcoma (AS) is a low-grade malignancy that is rare in premenopausal women. AS is a biphasic tumor with a benign epithelial component and a low-grade malignant sarcomatous component. Two histologic characteristics appear to have a poorer prognosis in AS: sarcomatous overgrowth (SO) and the presence of heterologous elements. These characteristics are found in about 10–15% of cases (Togami et al., 2018, Nannini et al., 2018, Verschraegen et al., 1998). The stromal component of the neoplasm may include uterine elements (homologous) or differentiate towards elements not typically found in the uterus (heterologous). Typically, rhabdomyosarcomatous differentiation is the most common (Patrelli et al., 2011). SO is defined as pure sarcomatous histology seen in at least 25% of the tumor. ASSO is aggressive and often associated with postoperative recurrence, metastatic disease, and fatal outcomes (Patrelli et al., 2011). In a gynecologic oncology group study of 31 cases of AS, 17 were diagnosed with SO on final pathology. Forty-four percent of ASSO patients recurred, and 31% died of their disease compared to 14% and 7% without SO, respectively (Tse et al., 2011). In their review of 19 AS cases, Tanner et al. concluded that both the 2-year progression-free survival (PFS) and overall survival (OS) of ASSO were statistically significant at 20% versus 100% for patients without SO (Tanner et al., 2013). Additional AS histological and staging characteristics have been reported as seemingly poor prognostic factors. These characteristics include high mitotic rate, cytological atypia, deep myometrial invasion, necrosis, and extrauterine spread (Togami et al., 2018, Kudela et al., 2019, Park et al., 2004).

The presenting symptoms of AS can vary, although abnormal vaginal bleeding is the most common in the literature review. Verschraegen et al. (1998) reported chief complaints of abnormal vaginal bleeding accounted for 71% of cases reviewed. Additional presenting signs and symptoms included pelvic mass (37%), uterine polyps (22%), and enlarged uterus (22%) (Verschraegen et al., 1998). Some studies also include foul vaginal discharge, as seen in the case presented (Togami et al., 2018, Kudela et al., 2019). Physical exam and imaging findings are typically similar to that of benign leiomyomas. In a literature review, most cases, including those with AS of the cervix, presented with a polypoid mass protruding through or extending from the endocervical canal (Patrelli et al., 2011, Park et al., 2004, Manoharan et al., 2007). This is similar to what was seen on physical examination of the case presented, where a 21 cm mass in total had extended through the cervical canal and into the vagina.

Presently, there is no reliable way to differentiate a benign versus malignant uterine leiomyoma before histopathologic review. MRI findings that may suggest AS of the uterus include marked enlargement of the uterus, myometrial thinning, polypoid mass protruding from the endometrial cavity, and mass that contains solid components with high signal intensity (Wang et al., 2010). Endometrial biopsy is typically performed in patients with risk factors or symptoms suggesting malignant neoplasm. A biopsy is also indicated in a prolapsed mass if the appearance is not consistent with a benign-appearing lesion, even in premenopausal women.

In a review of risk factors for uterine AS, a few proposed are currently discussed, including a history of pelvic irradiation, hyperestrogenism, and history of tamoxifen use (Patrelli et al., 2011). Mubeen et al. reported a compelling case of a 45 year old who previously received whole pelvic radiotherapy for squamous cell carcinoma of the cervix 20 years before her diagnosis of uterine ASSO (Mubeen et al., 2020). Other literature also suggests obesity, diabetes mellitus, endometriosis, and adenomyosis as possible risk factors (Wang et al., 2010). The only significant risk factor for the patient presented was 8 months of oral contraceptive use. Wang et al. additionally recommend considering frequent ultrasounds in patients that have high-risk features (Wang et al., 2010). As previously mentioned, there are no definitive ways to determine malignant potential without meticulous histologic examination. Therefore, high suspicion is required to diagnose and treat adequately.

The mainstay of treatment of AS involves surgical resection; however, the role and benefit of adjuvant chemotherapy remain undefined in ASSO. Currently, there are no clear treatment guidelines for patients with ASSO and localized disease. Most notably, in their single-center review of treatment responses in 7 patients with AS, Nannini et al. discussed a 60-year-old female with stage IB uterine ASSO with less than 50% myometrial invasion and no LVSI. She did not receive any adjuvant therapy, similar to this presented case. She was without evidence of disease at 84 months (Nannini et al., 2018). There was a questionable recurrence on lung biopsy in the case presented; however, the final surgical pathology was benign. The significance of her lung biopsy results is unclear, but she has continued to be free of disease since that time. In contrast, Nannini et al. reported another case of stage IC uterine ASSO with over 50% myometrial invasion. The patient recurred in the lungs despite adjuvant Doxorubicin. At the most recent follow-up, she was negative for disease at 32 months (Nannini et al., 2018). These cases raise the question of the significance of the depth of myometrial invasion in relation to the risk of disease recurrence.

Additionally, Tanner et al. performed a retrospective analysis of 31 uterine AS patients, 19 of which were treated from their initial diagnosis. Five out of 19 patients demonstrated SO. All 5 ASSO patients received surgical resection, three of the 5 cases contained heterologous elements, and two of 5 had myometrial invasion. Both of the patients with myometrial invasion had a recurrence. However, two of the remaining 3 patients without myometrial invasion also recurred (Tanner et al., 2013). The authors concluded that myometrial invasion was the most common high-risk feature for recurrent disease, seen in 31% of cases, followed by heterologous elements, SO, and residual disease at the time of initial surgery at 23%, 23%, and 8%, respectively (Tanner et al., 2013). It is possible that despite the presence of SO in the presented case, the patient may remain disease-free at this time because of the lack of significant myometrial invasion. Additionally, based on this study, heterologous elements were associated with an increased risk of recurrence. Nonetheless, the patient presented in this case remains disease-free despite the presence of this poor prognostic factor.

Ultimately, the patient presented in this paper did not receive adjuvant therapy and has been in remission since the initial surgery for 19 months. Although there is no clear consensus on adjuvant treatment based on the staging of disease, it should be considered that early-stage ASSO may be amenable to radical surgical resection alone in young patients. Some studies of cervical ASSO report impressive disease-free survival in young patients with confined disease who only received surgical resection (Park et al., 2004, Manoharan et al., 2007). In contrast, Togami argued that fertility preservation with local excision might be a reasonable option in patients without SO only. In their study of 6 patients with cervical adenosarcoma, 2 patients presented with SO. One of these patients underwent total abdominal hysterectomy and bilateral salpingo-oophorectomy without adjuvant therapy; they did not have any stromal invasion or heterologous elements on the final pathology. That patient had a lung recurrence 60 months post-operatively and succumbed to the disease 108 months after the initial surgery (Togami et al., 2018). Hanyuan Liu et al. reported a case of a 16 year old with uterine adenosarcoma, completely removed with hysteroscopy, who refused a hysterectomy. She ultimately had a recurrence in the uterus 7 months later and underwent total hysterectomy with ovarian preservation, final pathology with SO and heterologous differentiation, pathologic stage IC. The patient underwent 5 cycles of gemcitabine and docetaxel, and she has been without disease at follow-ups since treatment (Liu et al., 2018). This suggests that local excision of uterine ASSO alone is insufficient; however, management with radical tumor debulking and achieving negative margins may have a favorable outcome in young early staged patients.

Further studies must be conducted on uterine ASSO to determine the appropriate course of postoperative treatment dependent on the stage and histologic elements. Nonetheless, given the aggressive nature of ASSO, early diagnosis and treatment are mandatory for a favorable outcome. In patients such as the one examined in this paper who have solely undergone radical surgical resection in early-stage disease, achieving negative margins is mandatory. Additionally, close surveillance with imaging and exams seems a reasonable option as she remains disease-free for 19 months.

### CRediT authorship contribution statement

**D.D. Dowding:** Writing – original draft, Writing – review & editing, Project administration. **L.M. Wayne:** Writing – original draft, Writing – review & editing. **A.S. Guirguis:** Conceptualization, Writing – review & editing, Supervision.

## Declaration of Competing Interest

The authors declare that they have no known competing financial interests or personal relationships that could have appeared to influence the work reported in this paper.
