# Supplementary figures and images for: High grade uterine adenosarcoma with sarcomatous overgrowth in a young woman amenable to primary surgical reduction: A case study and literature review
Source: Gynecol Oncol Rep. 2021 Dec 29;39:100920. doi: 10.1016/j.gore.2021.100920 (PMC8749193; doi:10.1016/j.gore.2021.100920)

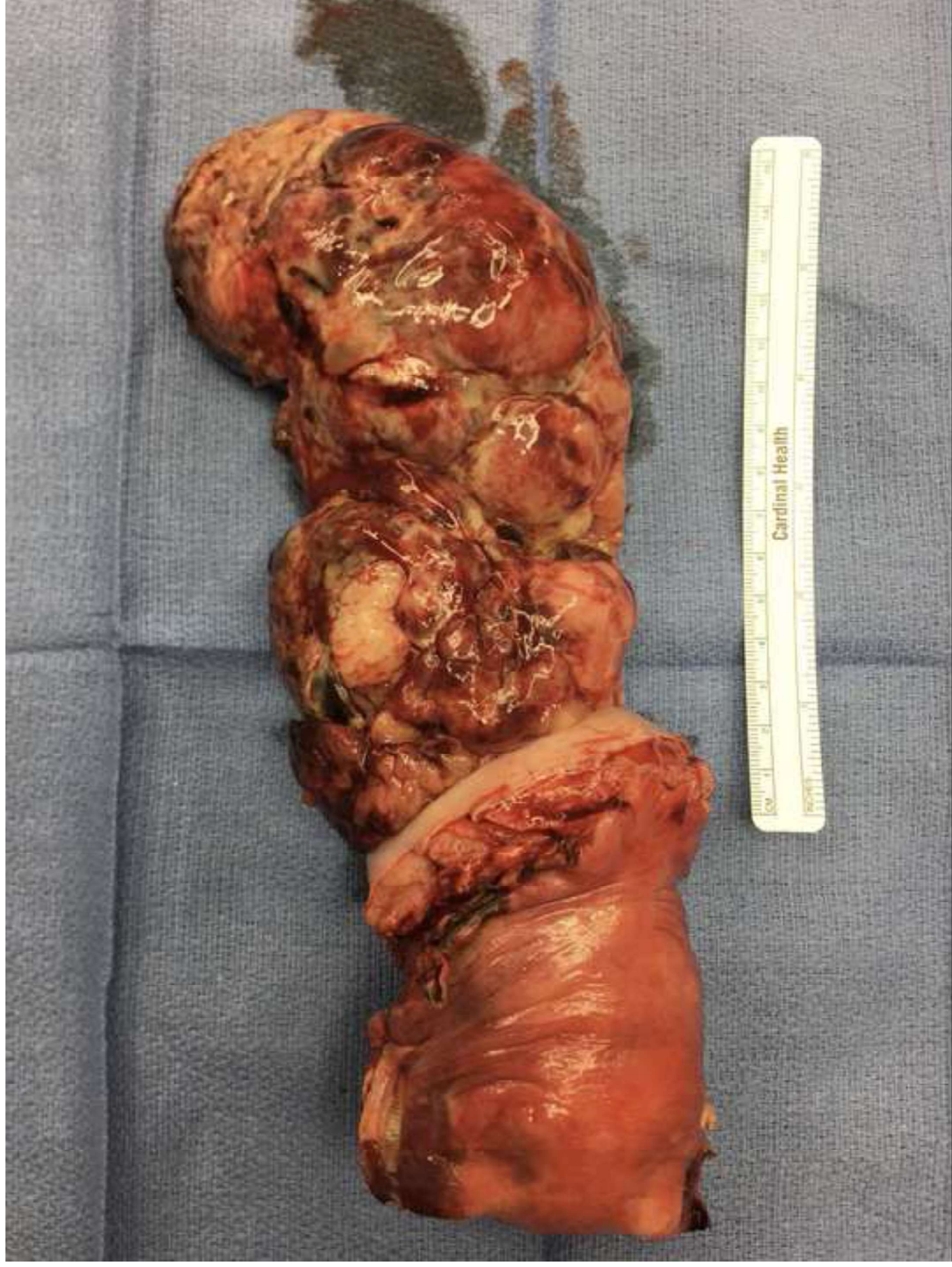

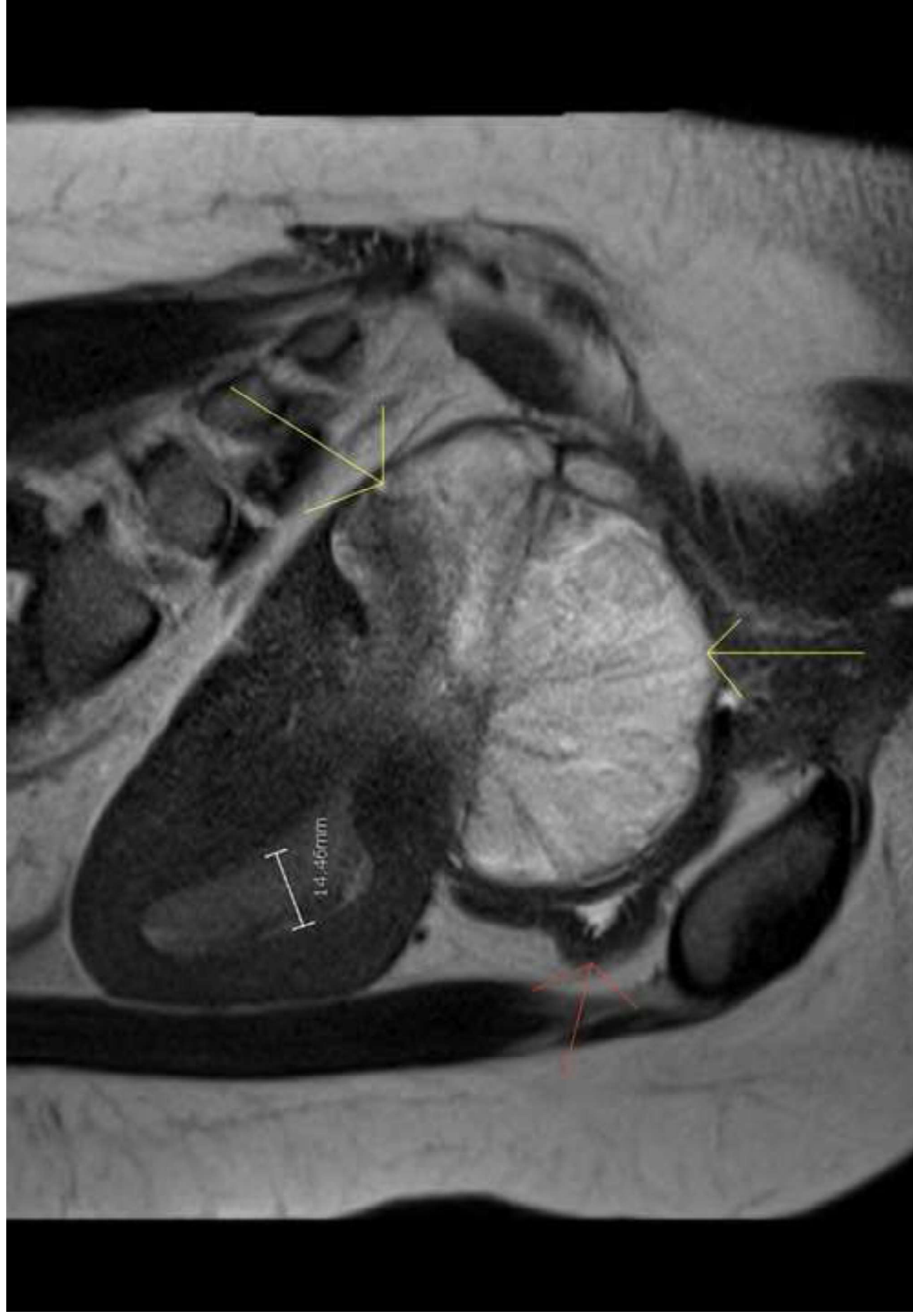

Supplement: Supplementary data 1 [file mmc1.pdf]
